# Knowledge, Attitude, and Practice Regarding Media Reporting on Suicide among Media Persons of a Province

**DOI:** 10.31729/jnma.8000

**Published:** 2024-02-29

**Authors:** Madhur Basnet, Prekshya Thapa, Dhana Ratna Shakya, Suraj Nepal, Neena Rai, Kailash Khaki Shrestha

**Affiliations:** 1 Department of Psychiatry, B.P. Koirala Institute of Health Sciences, Dharan, Sunsari, Nepal; 2 Department of Psychiatric Nursing, College of Nursing, B.P. Koirala Institute of Health Sciences, Dharan, Sunsari, Nepal; 3 Department of Psychiatry, KIST Medical College and Teaching Hospital, Mahalaxmi, Lalitpur, Nepal; 4 Department of Public Health, Edenburg International College, Purbanchal University, Biratnagar, Morang, Nepal

**Keywords:** *attitude*, *cross-sectional study*, *knowledge*, *suicide*

## Abstract

**Introduction:**

Suicide is a global public health problem. Sensible media reporting on suicide could be useful in suicide prevention. This study aimed to evaluate the knowledge, attitude, and practice regarding media reporting on suicide among media persons of a province.

**Methods:**

A qualitative study was done among media persons of the province after obtaining ethical approval from the Institutional Review Committee. Data was collected through online platform using Google form from 15 April 2022 and 15 January 2023. A convenience sampling method was used. Descriptive statistics were used for the data analysis.

**Results:**

Among 165 media persons, 54 (32%) of the participants either agreed to or were neutral about prominently reporting suicide news, and 47 (28.48%) were undecided or agreed about mentioning the details of the event. Only 50 (30.30%) thought that it is always possible to help a person with suicidal thoughts. A total of 48 (29.09%) always reported providing information about where to seek help in case one is suicidal.

**Conclusions:**

Media personnel were found to possess better knowledge about suicide reporting but exhibited inadequate practice in terms of providing method and site details and promoting support services, alongside maintaining an unfavorable attitude towards suicide. There is an urgent need to focus on coordination, standardisation, evidence generation and capacity building of media persons on suicide.

## INTRODUCTION

Suicide, an act of intentional killing of oneself, is a global public health problem with an estimated 8,00,000 people dying due to suicide every year globally.^1^ Scientific evidence worldwide demonstrates that media reporting of suicide can result in contagion, with increased suicide rates across the population.^2,3^

There is some evidence that reporting of suicide in a few Asian countries is more graphic, explicit, and simplistic than in Europe and the United States.^4,5^ Most reports in Nepal provided specific details about the location, the specific method used, posted the picture, and other details. This is far removed from the World Health Organization's (WHO) recommendations for covering such news.^6^ In countries like Nepal, the news content is heavily influenced by individual knowledge and attitude of the media person.^7^

This study aimed to evaluate the knowledge, attitude, and practice regarding media reporting on suicide among media persons of a province.

## METHODS

A qualitative study was done among media persons of Province 1 from 15 April 2022 and 15 January 2023. Ethical approval was obtained from the Institutional Review Committee of B. P. Koirala Institute of Health Sciences, Dharan, Nepal (Reference number: IRC/2249/022). Data was collected through online platform using Google form. Form link was sent through SMS, WhatsApp, or email as appropriate to the media persons. The participants were provided with the participant information sheet on the first page of the Google form and asked for their voluntary willingness to participate in the study. They were then directed to the enrollment in the study only once they clicked on the "Yes" button at the end of the page. A list of media persons-news reporters and editors aged 18 years and above who were actively working in Province 1 was prepared by collecting information through the Federation of Nepalese Journalists, Province 1 committee, and personal contact with the media persons and included in the study. Those who were inactive in the media for the past six months were excluded from the study. The form was sent two more times for those who did not respond over one month. A purposive sampling method was used. Authors have sent the Google forms to 215 participants. A total of 165 participants responded to the form in a total of three attempts making a response rate of 77%.

The authors prepared a semi-structured questionnaire after reviewing literature for the collection of socio-demographic profiles, knowledge, and practice regarding media reporting on suicide and the participants' felt the need for further support/training. There are no validated standard instruments to assess the knowledge and practice of suicide media reporting so we prepared one by ourselves primarily based on the "Preventing Suicide: A Resource for Media Professionals, update 2017" published by the WHO and discussed with the experts.^1^ The questionnaire on knowledge about suicide media reporting consisted of 12 questions and on practice consisted of 14 questions. Each question on knowledge had responses on a five-point Likert scale ranging from "strongly agree" to "strongly disagree" while those in the practice questionnaire had responses on a five-point Likert scale ranging from "always" to "never". The questions were pretested among five media persons and refined further to ensure content validity. The Attitude Towards Suicide Scale (ATTS) was used to examine the attitude towards suicide among the media persons. This scale is available in English language and was translated into Nepali language using the standard protocol for language translation. It contains 37 items with a five-point Likert scale from "strongly agree" to "strongly disagree".^12^

Authors collapsed the "strongly agree/agree" responses into "agree" and "strongly disagree/disagree" into "disagree" to see if favourable or unfavourable attitudes were better. For attitude section, authors tried to analyze responses in various factors depending on their domain-acceptability of suicide (items 5, 16, 16, 18, 20, 26, 29, 31, 32, 36), preventability/preparedness to prevent (items 6, 9, 21, 30, 37), tabooing (items 3, 11, 12, 13, 23, 24, 33), relationship issues/cause (items 7, 8, 14, 25, 35), incomprehensibility (items 2, 27, 28), process (items 4, 10), unpredictability (items 17, 22) and suicide as a right (item 34). A Google form was created with the sociodemographic profile and the questionnaires on knowledge, attitude, and practice in the Nepali language. It was sent to the participants which was to be self-administered by them.

Data was entered in Microsoft Excel 2019 and analysed using IBM SPSS Statistics version 22.0. Descriptive statistics were used for data analysis.

## RESULTS

Among 165 respondents, the majority of the participants were 136 (82.42%) male, Hindu 115 (69.69%), 132 (80%) Terai districts and had education 125 (75.75%) of bachelor level and above. The mean age of the participants was 35.7±8.5 years with an age range of 20-70 years and the mean duration of engagement in the media was 13.6±7.4 years. The mean number of times suicides were reported/edited in media in the last year was 2.7±3.9 (Table 1).

**Table 1:** Sociodemographic profile (n = 165).

Variable	Category	n (%)
Type of Media	Print Media	22 (13.33)
	Online Media	27 (16.36)
	Radio/TV-broadcasting media	35 (21.21)
	Multiple	81 (49.09)
Role in Media	Reporter	64 (38.79)
	Editor	60 (36.36)
	Both	41 (24.85)
Personal experience of the death of some close acquaintance by suicide		122 (73.94)
Suicidal ideas in the last one month		10 (6.06)
Suicidal plan in lifetime		16 (9.70)
Suicidal attempt in lifetime		8 (4.85)
Training on responsible media reporting on suicide		19 (11.52)

Almost one-third 54 (32.73%) of participants either agreed or were neutral about placing suicide-related stories prominently and unduly repeating such stories while almost half 76 (46.06%) were either undecided or agreed about mentioning the details of the site of the event (Table 2).

**Table 2:** Knowledge regarding media reporting on suicide (n = 165).

S.N.	Questions	Strongly Agree	Agree	Neutral	Disagree	Strongly Disagree
1.	Provide accurate information about where to seek help in case one is suicidal	76 (47.27)	60 (36.36)	19 (11.52)	6 (3.64)	2 (1.21)
2.	Educate the public about the facts of suicide and suicide prevention	136 (82.42)	34 (20.61)	1 (0.61)	2 (1.21)	2 (1.21)
3.	Report stories of how to cope with life stressors or suicidal thoughts, and how to get help	104 (63.03)	54 (32.73)	4 (2.42)	3 (1.82)	-
4.	Apply particular caution when reporting celebrity suicides	83 (50.30)	68 (41.21)	4 (2.42)	7 (4.24)	3 (1.82)
5.	Apply caution when interviewing bereaved family or friends	135 (81.82)	27 (16.36)	-	-	3 (1.82)
6.	Media professionals themselves may be affected by stories about suicide	96 (58.18)	58 (35.15)	3 (1.82)	6 (3.64)	2 (1.21)
7.	Place stories about suicide prominently and unduly repeat such stories	16 (9.70)	26 (15.76)	12 (7.27)	77 (46.67)	34 (20.61)
8.	Use language which sensationalizes or normalizes suicide, or presents it as a constructive solution to problems	22 (13.33)	26 (15.76)	5 (3.03)	30 (18.18)	82 (49.70)
9.	Explicitly describe the method used	12 (7.27)	17 (10.30)	18 (10.91)	59 (35.76)	59 (35.76)
10.	Provide details about the site/location	17 (10.30)	40 (24.24)	19 (11.52)	59 (35.76)	30 (18.18)
11.	Use sensational headlines	9 (5.45)	3 (1.82)	6 (3.64)	60 (36.36)	87 (52.73)
12.	Use photographs, video footage or social media links	9 (5.45)	3 (1.82)	4 (2.42)	75 (45.45)	74 (44.85)

There was unfavorable attitude towards the acceptability of suicide however there were favorable attitude towards suicide in the majority of items of domains like preventability e.g. human duty to help a suicidal person 162 (98.18%), prepared to help a person in suicidal crisis 129 (78.18%) suicide can be prevented 152 (92.12%) and relationship issues/cause e.g. people who commit suicide are usually mentally ill 75 (45.45%) and disagreement that many suicide attempts are made to take revenge 97 (58.79%) (Table 3).

**Table 3:** Attitude towards suicide as per the attitude towards suicide scale (n = 165).

S.N.	Description	Agree n (%)	Undecided n (%)	Disagree n (%)
1.	It is always possible to help a person with suicidal thoughts.	50 (30.30)	66 (40)	49 (29.70)
2.	Suicide can never be justified.	147 (89.09)	10 (6.06)	8 (4.85)
3.	Committing suicide is among the worst things to do to one's relatives.	102 (61.82)	28 (16.97)	35 (21.21)
4.	Most suicide attempts are impulsive actions.	136 (82.42)	18 (10.91)	11 (6.67)
5.	Suicide is an acceptable means to terminate an incurable disease.	16 (9.70)	20 (12.12)	129 (78.18)
6.	Once a person has made up his/her mind about committing suicide no one can stop him/her.	8 (4.85)	32 (19.39)	125 (75.76)
7.	Many suicide attempts are made because of revenge or to punish someone else.	31 (18.79)	37 (22.42)	97 (58.79)
8.	People who commit suicide are usually mentally ill.	75 (45.45)	23 (13.94)	67 (40.61)
9.	It is a human duty to try to stop someone from committing suicide.	162 (98.18)	1 (0.61)	2 (1.21)
10.	When a person commits suicide, it is something that he/she has considered for a long time.	80 (48.48)	48 (29.09)	37 (22.42)
11.	There is a risk of evoking suicidal thoughts in a person's mind if you ask about it.	71 (43.03)	46 (27.88)	48 (29.09)
12.	People who make suicidal threats seldom complete suicide.	70 (42.42)	47 (28.48)	48 (29.09)
13.	Suicide is a subject that one should rather not talk about.	54 (32.73)	12 (7.27)	99 (60)
14.	Loneliness could for me be a reason to take my life.	44 (26.67)	37 (22.42)	84 (50.91)
15.	Almost everyone has at one time or another thought about suicide.	63 (38.18)	46 (27.88)	56 (33.94)
16.	There may be situations where the only reasonable resolution is suicide.	35 (21.21)	42 (25.45)	88 (53.33)
17.	I could say that I would take my life without actually meaning it.	21 (12.73)	51 (30.91)	93 (56.36)
18.	Suicide can sometimes be a relief for those involved.	21 (12.73)	47 (28.48)	97 (58.79)
19.	Suicides among young people are particularly puzzling since they have everything to live for.	103 (62.42)	38 (23.03)	24 (14.55)
20.	I would consider the possibility of taking my life if I were to suffer from a severe, incurable, disease.	53 (32.12)	36 (21.82)	76 (46.06)
21	A person once they have suicidal thoughts will never let them go.	15 (9.09)	39 (23.64)	111 (67.27
22	Suicide happens without warning.	81 (49.09)	42 (25.45)	42 (25.45)
23.	Most people avoid talking about suicide.	114 (69.09)	24 (14.55)	27 (16.36)
24.	If someone wants to commit suicide it is their business and we should not interfere.	7 (4.24)	8 (4.85)	150 (90.91)
25.	It is mainly loneliness that drives people to suicide.	91 (55.15)	29 (17.58)	45 (27.27)
26.	A suicide attempt is essentially a cry for help.	55 (33.33)P	62 (37.58)	48 (29.09)
27.	On the whole, I do not understand how people can take their lives.	117 (70.91)	27 (16.36)	21 (12.73)
28.	Usually, relatives have no idea about what is going on when a person is thinking of suicide.	112 (67.88)	31 (18.79)	22 (13.33)
29.	A person suffering from a severe, incurable, disease expressing wishes to die should get help to do so.	27 (16.36)	27 (16.36)	111 (67.27)
30.	I am prepared to help a person in a suicidal crisis by making contact.	129 (78.18)	13 (7.88)	23 (13.94)
31.	Anybody can commit suicide.	115 (69.70)	29 (17.58)	21 (12.73)
32.	I can understand that people suffering from a severe, incurable, disease commit suicide.	70 (42.42)	65 (39.39)	30 (18.18)
33.	People who talk about suicide do not commit suicide.	14 (8.48)	72 (43.64)	79 (47.88)
34.	People do have the right to take their own lives.	20 (12.12)	17 (10.30)	128 (77.58)
35.	Most suicide attempts are caused by conflicts with a close person.	81 (49.09)	37 (22.42)	47 (28.48)
36.	I would like to get help to commit suicide if I were to suffer from a severe, incurable, disease.	20 (12.12)	32 (19.39)	113 (68.48)
37.	Suicide can be prevented.	152 (92.12)	10 (6.06)	3 (1.82)

A total of 59 (35.75%) reported occasionally or never providing information about where to seek help while 42 (25.45%) reported not knowing where to seek help. A total of 21 (12.72% reported not knowing about the stories of how to cope with life stressors or suicidal thoughts, and how to get help and 11 (6.67%) reported not applicable for applied particular caution when reporting celebrity suicides. More than one-third 58 (35.15%) reported always or often providing details about the site/location of the event (Table 4).

The majority 127 (76.96%) of the participants either disagreed or were undecided regarding the adequacy of their knowledge and practice regarding media reporting on suicide and so was the felt lack of access to resources 126 (76.36%). The majority of the participants 85 (51.52%) strongly agreed and another 66 (40%) agreed on the need for further training on responsible media reporting on suicide.

**Table 4:** Practice regarding media reporting on suicide (n = 165).

S.N.	Questions	Always	Most of the time	Often	Occasionally	Never
1.	Provided information about where to seek help in case one is suicidal	48 (29.09)	13 (7.88)	3 (1.82)	25 (15.15)	34 (20.61)
2.	Provided information to the public about the facts of suicide and suicide prevention in your report	51 (30.91)	48 (29.09)	3 (1.82)	54 (32.73)	9 (5.45)
3.	Mentioned stories of how to cope with life stressors or suicidal thoughts, and how to get help	44 (26.67)	41 (24.85)	8 (4.85)	39 (23.64)	12 (7.27)
4.	Applied particular caution when reporting celebrity suicides	101 (61.21)	26 (15.76)	4 (2.42)	15 (9.09)	8 (4.85
5.	Applied caution when interviewing bereaved family or friends	132 (80)	18 (10.91)	1 (0.61)	8 (4.85)	6 (3.64)
6.	Placed stories about suicide prominently in front page	6 (3.64)	5 (3.03)	4 (2.42)	37 (22.42)	113 (68.48)
7.	Repeatedly reported news about same suicide	3 (1.82)	3 (1.82)	2 (1.21)	34 (20.61)	122 (73.94)
8.	Used sensational language to attract public attention	4 (2.42)	1 (0.61	3 (1.82)	11 (6.67)	146 (88.48)
9.	Used language portraying suicide as an escape from stresses in life	5 (3.03)	1 (0.61)	1 (0.61)	7 (4.24)	151 (91.52)
10.	Used language portraying suicide as a normal/usual event	32 (19.39)	8 (4.85)	3 (1.82)	11 (6.67)	111 (67.27)
11.	Explicitly described the method used while committing suicide	30 (18.18)	11 (6.67)	4 (2.42)	30 (18.18)	90 (54.55)
12.	Provided details about the site/location of the suicide	40 (24.24)	13 (7.88)	5 (3.03)	32 (19.39)	75 (45.45)
13.	Used sensational headlines or mentioned "Suicide" in the headlines	36 (21.82)	1 (0.61)	1 (0.61)	7 (4.24)	120 (72.73)
14.	Used photographs, video footage or social media links	34 (20.61)	3 (1.82)	-	16 (9.70)	122 (73.94)

## DISCUSSION

The gender distribution of Province 1 as per the national census of 2011 was 52% females while females were grossly underrepresented (17.6%) among media persons in our study.^8^ Similarly, Brahmin (35.2% vs 12%) and Chhetri (19.4% vs 15%) were highly overrepresented in the media in Province 1.8 Suicidal ideation in the last month in our study was the same (6.1%) as that of the national average as per the national mental health survey (6.1%) however, suicidal attempt ever was almost five times higher (4.8%) than the national report (1.1%).^9^ This might be because media persons are at higher risk of exposure to traumatic events and higher stress in the profession as well. This is an alarming situation and needs prompt intervention.

Overall, there was good knowledge regarding responsible media reporting on suicide among the media persons in our study despite only 19 (11.5%) of them reporting having had training on the matter. However, there was relatively less knowledge regarding providing details of the site with almost one-third reporting "agree/strongly agree". Similarly, there was relatively less knowledge regarding the method used and using language that sensationalizes, normalizes, or presents suicide as a solution to problems.

Regarding the practice, almost one-fourth (25.5%) reported not knowing about the source of seeking help when one is suicidal while almost another one-third reported occasionally or never mentioning it while reporting. This is similar to the findings in a study from Ireland where 75.8% did not refer to appropriate support services for people vulnerable to, and at risk of suicide,^10^ and that from India where only 2.5% gave contact details for a suicide support service.^4^ As providing information on suicide prevention support services could help prevent suicide among those at risk, this could be a major area of intervention regarding responsible media reporting on suicide.^11^ Almost one-third of the participants reported providing details of the method of suicide from "occasionally to always" which is similar to the findings (27.3%) of a study on the quality of media reporting on suicide from Nepal.^12^ As mentioned suicide method in the news could increase the risk of suicide among those contemplating it, this could be another issue to address.^11^ Only 8 (4.8%) of the participants reported repeating the same suicide news "occasionally to always" in our study which is much better than the findings from India with 68.6% of the news being repeated.^4^

Regarding attitude towards suicide, overall, there was an unfavourable attitude towards suicide. As the attitude of the journalists towards suicide has been found to affect the adherence to suicide media reporting guidelines this area needs to be addressed well.^13^ The majority of the participants disagreed with the adequacy of their knowledge and good access to resources on responsible media reporting on suicide and their knowledge and practice also reflect their felt need. As responsible media reporting of suicides has been identified as a promising and potentially effective population-level suicide prevention intervention addressing these issues could be a major area of intervention regarding suicide prevention in Nepal.^14,15^

One of the major limitations of this study was the higher non-response rate eg. 23%. However, this is usual in online questionnaire-based studies. Similarly, as this study consisted of self-administered questionnaires, this could reduce the honesty and accuracy of the responses as compared to interviewer-administered questionnaires. However, this could also be a strength as participants could be more comfortable on sensitive issues like suicide reporting and suicidal ideas through a self-administered questionnaire than in face-to-face interviews. The generalizability of the study could not be done due to the small sample size and use of the convenience sampling method.

## CONCLUSIONS

The knowledge regarding media reporting on suicide was found to be better among the media persons but their practice was lacking in many of the aspects like providing details of the method and site and providing information about support services for those at risk. Similarly, there was an overall unfavourable attitude towards suicide. To improve responsible media reporting on suicide in Nepal, training and education should be provided to media professionals on avoiding sensationalism and providing accurate information about support services. Additionally, efforts should be made to shift the attitudes of media professionals towards suicide, emphasizing the importance of responsible reporting and its impact on vulnerable populations.

## References

[1] World Health Organization. (2019). Suicide worldwide in 2019: global health estimates [Internet]..

[2] Sinyor M, Schaffer A, Heisel MJ, Picard A, Adamson G, Cheung CP (2018). Media guidelines for reporting on suicide: 2017 update of the Canadian psychiatric association policy paper.. Can J Psychiatry..

[3] Tatum PT, Canetto SS, Slater MD (2010). Suicide coverage in U.S. newspapers following the publication of the media guidelines.. Suicide Life Threat Behav..

[4] Armstrong G, Vijayakumar L, Niederkrotenthaler T, Jayaseelan M, Kannan R, Pirkis J (2018). Assessing the quality of media reporting of suicide news in India against World Health Organization guidelines: a content analysis study of nine major newspapers in Tamil Nadu.. Aust N Z J Psychiatry..

[5] Arafat SMY, Kar SK, Marthoenis M, Cherian A V., Vimala L, Kabir R (2020). Quality of media reporting of suicidal behaviors in South-East Asia.. Neurol Psychiatry Brain Res..

[6] World Health Organization (WHO). (2017). Preventing suicide: a resource for media professional 2017 [Internet]..

[7] Marahatta K, Samuel R, Sharma P, Dixit L, Shrestha BR (2017). Suicide burden and prevention in Nepal: The need for a national strategy.. WHO South East Asia J Public Health..

[8] Province 1 Data Summary. National Data Portal-Nepal [Internet]..

[9] Nepal Health Research Council. (2020). National mental health survey, Nepal: 2020 factsheet (adults) [Internet]..

[10] McTernan N, Spillane A, Cully G, Cusack E, O'Reilly T, Arensman E (2018). Media reporting of suicide and adherence to media guidelines.. Int J Soc Psychiatry..

[11] Niederkrotenthaler T, Voracek M, Herberth A, Till B, Strauss M, Etzersdorfer E (2010). Role of media reports in completed and prevented suicide: Werther v. Papageno effects.. Br J Psychiatry..

[12] Sharma P, Timasina RR, Singh S, Gyawali S, Marahatta K, Arafat SMY (2022). Quality of media reporting of suicide in Nepal.. Psychiatry J.

[13] Eimontas J, Gailiene D (2014). Attitudes of journalists who write about suicide towards guidelines for reporting on suicide and both favourable and unfavourable factors for compliance with the guidelines.. Vilnius University Press..

[14] Zalsman G, Hawton K, Wasserman D, van Heeringen K, Arensman E, Sarchiapone M (2016). Suicide prevention strategies revisited: 10-year systematic review.. Lancet Psychiatry..

[15] Turecki G, Brent DA (2016). Suicide and suicidal behaviour.. Lancet..

